# Dietary and physical trigger factors in hereditary angioedema: Self-conducted investigation and literature overview 

**DOI:** 10.5414/ALX02523E

**Published:** 2024-11-14

**Authors:** Julia Zarnowski, Regina Treudler

**Affiliations:** 1 Department of Dermatology, Venereology and Allergology, University Hospital Leipzig, Leipzig, and; 2 Institute of Allergology, Charité University Hospital Berlin, Campus Benjamin Franklin, Berlin, Germany

**Keywords:** hereditary angioedema, trigger, food, nutrition, diet, intolerance, sports, fitness, leisure, lifestyle, burden, quality of life

## Abstract

Background: In hereditary angioedema (HAE), numerous factors are known to trigger an attack. The possible influence of diet or recreational sports has been given little consideration in studies. The aim of our work was to investigate the influence of nutrition and physical activity in patients with HAE at the Leipzig ACARE Center. Materials and methods: Patients with HAE were given a self-designed questionnaire inquiring for family history, disease progression, and encountered burden due to HAE, current therapy, and disease control (angioedema control test (AECT)) as well as the influence of diet and/or recreational sports on HAE attacks. Results: Inclusion of 30 patients (23 female, 77%) with a mean age of 49.5 ± 16.9 years and mean body mass index of 25.1 ± 6.4 kg/m^2^. 60% received prophylactic treatment, and 37% received exclusively on-demand therapy. The mean AECT score was 10.9 ± 5.1 and patients reported 15.5 ± 26.9 days of absence due to HAE attacks in the last 12 months. 33% reported an association with food intake, in particular worsening of abdominal symptoms (n = 7), swelling of the extremities (n = 3), face, larynx, or genital area (n = 1 each). 70% reported regular exercise, most commonly cycling (n = 11), running or walking (n = 10), or strength training (n = 10). 62% reported a worsening of HAE due to recreational exercise. Conclusion: Dietary factors and physical activity frequently led to an aggravation of HAE in our cohort and should be taken into consideration when counseling patients with regard to trigger avoidance.

## Introduction 

Hereditary angioedema (HAE) is a rare, autosomal dominant disease that often becomes symptomatic in the first to second decade of life. In most cases, the disease is caused by a deficiency or dysfunction of the enzyme C1 esterase inhibitor (C1-INH), which promotes an excess of bradykinin and leads to an increase in vascular permeability. Other genetic dysfunctions in bradykinin metabolism with normal C1-INH occur much less frequently. Clinically, there are recurrent (muco)cutaneous swellings that can occur on the skin as well as in the abdominal, urogenital, or laryngeal tissue and vary in duration, frequency, localization, and painfulness [1, 2, 3]. Due to the rarity and lack of awareness of HAE, missed or severely delayed diagnoses as well as unnecessary medication or medical interventions can occur [2, 4, 5, 6, 7]. 

Trigger factors that can cause HAE attacks include physical stress (mechanical impact, pressure), medication (ACE inhibitors, estrogen-containing contraceptives), infections, medical interventions and surgical procedures (e.g., dental surgery), psychological stress, or endogenous hormonal fluctuations (menstruation, menopause, pregnancy) [1, 3, 7, 8]. Numerous studies have demonstrated an increased socioeconomic burden due to HAE, with loss of productivity, increased sick days and negative effects on education and career [2, 4, 5, 9]. It has also been shown that HAE has a major impact on the daily life and leads to impairments in sleep quality, social life, travel behavior, and sporting activities [2, 4, 5, 10]. 

Pronounced restrictions in quality of life and high prevalence of mental illnesses such as depression and anxiety disorders have been described in patients with HAE. A significant association of HAE severity and control of disease was shown with psychiatric diseases, which is why early diagnosis and treatment are crucial [1, 3, 4, 10, 11, 12]. Therapeutically, medication for the acute treatment of attacks (C1-INH concentrates and the bradykinin B2 receptor antagonist icatibant) and for long-term prophylaxis (C1-INH concentrate, tranexamic acid, lanadelumab, berotralstat) are approved in Germany. A large number of other treatments are currently in development [13, 14, 15]. 

HAE affects patients on many levels, however, there are few studies in the current literature that investigate the effect of nutrition and recreational sports on HAE attacks. Diet and sports are playing an increasingly important role in patients’ daily living and quality of life. The aim of our questionnaire-based study was to investigate these little-studied trigger factors in HAE patients at the Department of Dermatology, University Hospital Leipzig, Germany. 

## Materials and methods 

Patients diagnosed with HAE were recruited from the allergology consultation at the Department of Dermatology, Venereology, and Allergology at University Hospital Leipzig (certified ACARE Center (Angioedema Center of Reference and Excellence [16])). After oral and written information and consent (ethics vote 115/19-ek), patients were given a questionnaire inquiring for demographic data, family history, current therapy, and disease control (angioedema control test (AECT)), the influence of diet and physical activity on HAE as well as experienced restrictions and burdens of HAE on private life, social activities, travel, and work. 

In addition, a PubMed-based literature search was conducted with the included search terms *“hereditary angioedema”* ± *“trigger” and food, nutrition, diet, intolerance, lifestyle, leisure, fitness, sports, physical activity* with a focus on the last 10 years (2014 – 2024). 

## Results 

30 patients were included, 23 of whom were female (77%), with an average age of 49.5 ± 16.9 years and an average body mass index of 25.1 ± 6.4 kg/m^2^. 

### Course of disease and therapy 

22 patients had HAE type I (quantitative C1-INH deficiency), 3 had type II (qualitative C1-INH deficiency), and 5 had HAE with normal C1-INH. 

The first symptoms of HAE occurred on average at 15.7 ± 7.3 years, but patients were diagnosed with HAE at 29.4 ± 15.4 years, resulting in a latency period of 13.7 ± 13.3 years between the first symptoms and the diagnosis. A total of 18/30 patients (60%) received prophylactic treatment and 12/30 (40%) received on-demand treatment only. The mean AECT score was 10.9 ± 5.1. On average, patients reported 15.5 ± 26.9 days of absence due to HAE attacks in the last 12 months. 

Within the past 12 months, n = 3 patients stated that they had been treated in the emergency room (1, 2, and 5 times, respectively), n = 1 patient had been treated as an inpatient (5 times in total), and n = 1 patient received intensive care treatment (once). 

During the entire duration of the illness, 29/30 patients stated that they received unplanned medical treatment due to an HAE attack, 23/29 in the emergency room (on average 26.3 ± 38.2 times in total), 22/29 as inpatients (on average 25.0 ± 39.0 times), and 8/29 in the intensive care unit (in 2/8 after a previous surgical procedure). 

### Family burden 

In 28/30 patients, other family members are affected by HAE (on average 4 relatives per patient) and in 7/30 family members died due to a laryngeal swelling; n = 3 patients had 1 or 2 deceased relatives and n = 1 patient had 3 deceased relatives. 7/30 stated that starting a family was subjectively more difficult due to HAE, of these only 1/7 had a deceased relative. 

### Allergological comorbidities and allergen immunotherapy 

9/30 patients reported on diagnosed allergies to inhalation or injection allergens, including animal hair (n = 2), tree pollen (n = 4), grass pollen (n = 4), wasp venom (n = 2). 2/9 reported a subjective worsening of HAE due to the known allergies (wasp and tree pollen, respectively). A total of 3/9 patients received allergen-specific immunotherapy (AIT) against grass pollen, tree pollen, and wasp venom, respectively. All patients (3/3) denied an influence of AIT on HAE symptoms. 

### Influence of diet on hereditary angioedema 

10/30 patients (33%) reported an observed relationship between food intake and HAE attacks, with an aggravation of symptoms after the consumption of milk (n = 1), legumes (n = 4), acidic foods (n = 1), stone fruit (n = 2), wheat-containing products (n = 2), and spicy foods (n = 1). Bloating foods were also reported by 2 patients (multiple answers possible). 

4/10 reported a known food allergy (n = 4 to legumes and n = 2 to pome fruit, multiple answers possible) and 3/10 a known food intolerance (n = 2 to lactose and n = 1 to sorbitol), each of which had a subjectively negative effect on HAE attacks. 

The most frequently reported food-associated HAE symptoms were abdominal symptoms (n = 7), swelling of the extremities (n = 3), or facial, laryngeal, and genital swelling (n = 1, respectively). 

### Influence of recreational sports on hereditary angioedema 

21/30 patients (70%) stated that they regularly practiced recreational sports, most frequently cycling (n = 11), running or hiking (n = 10, respectively), and strength training (n = 10), multiple answers were possible. 13/21 patients (62%) reported a worsening of HAE due to the recreational exercise. 6 patients reported an HAE attack due to mechanical pressure on palmoplantar areas during long hikes or strength training, 2 reported genital swelling after intensive cycling, and 1 patient reported an abdominal attack due to strong emotions (fear) during a climbing tour. 

### Subjective stress caused by hereditary angioedema 

**Figure 1.**
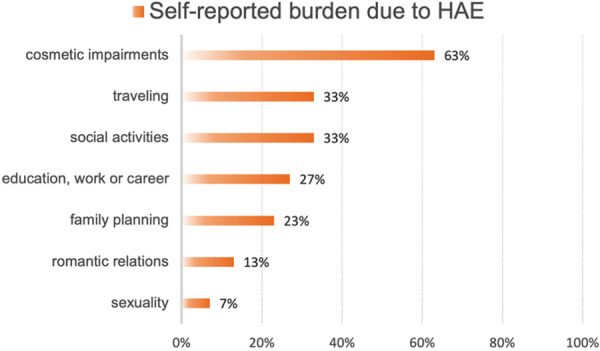
Self-reported  burden due to hereditary angioedema in different areas of life.

As shown in Figure 1, patients report on significant burden caused by HAE in various areas of life. 7% experienced a negative impact of HAE on sexuality, 13% on romantic relationships, 23% on family planning, and 27% on education or work. Moreover, 33% state a negative impact on social activities or travel and 63% experienced cosmetic impairments due to HAE-related swelling. In addition, 20/23 patients reported a previous pregnancy, of which 11 (55%) experienced a significant worsening of HAE frequency and intensity during gravidity. 

## Literature overview 

**Figure 2.**
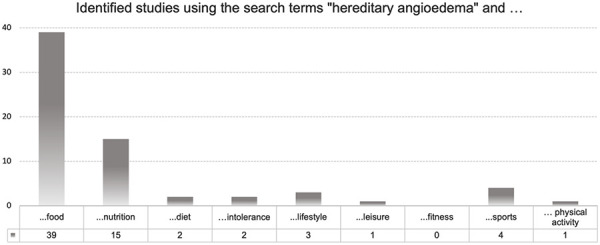
Identified studies using the search terms: “hereditary angioedema” and *food, nutrition, diet, intolerance, lifestyle, leisure, fitness, sports, physical activity*.

With regard to the literature research, only 1 study between 2014 and June 2024 could be found in PubMed using the search terms “hereditary angioedema”, “trigger” and the keywords “food”, “nutrition”, “intolerance”, and “sports”. The search term “trigger” was subsequently removed, so that a literature search was only carried out with “hereditary angioedema” and the keywords shown in Figure 2. 

### Limitation of the literature research 

It should be noted that 27 of the 39 studies with the search terms *“hereditary angioedema”* and *“food”* referred to the U.S. Food and Drug Administration (FDA) and not to foodstuff. Several studies had to be excluded because the terms *“sports”*, *“food”*, or *“nutrition”* were mentioned in the sections *Funding, Conflict of interest* or as part of the name of an *institution or database* and did not refer contextually to HAE. Numerous studies were excluded because the search terms were not mentioned as HAE triggers but used in a different context (e.g., *“physical activity”* as an impairment in HAE patients; *“food”* in the context of food anaphylaxis or pharmacokinetics). Finally, in almost all studies, the term *“nutrition”* referred to one of the four dimensions of the validated Angioedema Quality of Life Questionnaire (AE-QoL). Only a few studies that used the AE-QoL, address the relationship between nutrition and HAE, while most studies do not go into detail about the nutrition domain. 

### Literature review on hereditary angioedema, food, sports, and intolerances 

A Swedish study showed that the attack frequency or severity in HAE correlated significantly with a poorer evaluation of the AE-QoL nutrition domain, indicating a severe reduction in quality of life in this area. A greater burden in the nutrition dimension was also reported, if patients did not receive prophylactic therapy (37 versus 0 points; max. 100 points) [17]. Another study showed a significantly lower impairment of quality of life in the AE-QoL nutrition domains in patients treated prophylactically with lanadelumab [12]. 

A study of 42 HAE patients from Switzerland found that 79% were able to identify trigger factors for encountered attacks, most frequently emotions (79%), followed by trauma (55%), food (36%), and insect bites (6%) [18]. All patients who reported on foodstuff as HAE trigger suffered from abdominal attacks. They reported different fruits (citrus fruit, apple, kiwi, strawberry, pineapple, banana), vegetables (tomato, green salad, onion, garlic, leek), fish, shrimp, milk, chili, cheese, tree nuts, peanut, bread, or alcohol as potential triggers. However, the subsequent skin prick test for the accused foods was negative in all patients; in 11/12 patients, the determination of the specific IgE fx5 (food screening for chicken, egg white, milk protein, cod, wheat flour, peanut, and soy) and the IgEs for specific allergens were also negative. In addition, 3/12 patients suffered from lactose intolerance and reported a link between the consumption of dairy products and HAE attacks [18]. 

A study on trigger factors in 106 HAE patients showed that 23% (24/106) reported on foodstuff as triggers, such as coffee, alcohol, dairy products, spicy and fatty foods, legumes, nuts (walnuts), vegetables (tomato, onion, garlic), and gluten. Of these patients, 35% (8/24) stated to have a known food sensitization/allergy [19]. 

Other authors examined the HAE diaries of 92 patients at a Hungarian HAE center over a period of 7 years. The analysis showed that 70% reported physical exertion as the most common subjective trigger, and a total of 20% suspected a connection between the consumption of certain foods and an HAE episode. In addition, n = 2 patients cited “allergy” and n = 1 “excessive gastric acid secretion” as triggers. In the second part of the study, 27 HAE patients were followed up over a period of 7 months, during which daily diary entries were made about the encountered triggers and experienced attacks; overall 882 potential trigger factors and 365 attacks were recorded. In 9 cases, the consumption of food was indicated as a potential trigger, which also led to an HAE attack in 7/9 cases (7/7 abdominal) [8]. 

A retrospective study of 31 Swedish children found that abdominal attacks were most commonly caused by psychological stress, while trauma and sports were the most common triggers of cutaneous swelling [20]. Another study showed that the influence of trigger factors can be altered by the use of prophylactic therapy, as an example the frequency of sports-associated HAE attacks decreased and patients were able to resume sports that previously had to be abandoned [21]. In the same study, some patients also reported an improvement in appetite due to a reduction in abdominal attacks. Finally, individual patients stated that long-term prophylaxis enabled the consumption of certain foods that would have previously triggered an HAE attack [21]. 

With regard to the search term “intolerance”, one review aims to show the correlation between HAE and gluten intolerance or celiac disease. Screening for celiac disease and a gluten-free diet are recommended if abdominal symptoms persist in HAE patients despite an initiated therapy. The extent to which abdominal HAE attacks are triggered or improved by a gluten-rich or gluten-free diet is not explicitly investigated [22]. 

Using the more general search terms “leisure” and “lifestyle”, several studies were found that emphasize the need for individualized management plans, including emergency plans for travel, school and sports, as well as sufficient (prophylactic) therapy to ensure participation in school, work, family life, and leisure activities [23, 24]. 

## Discussion 

Our data illustrate the ongoing burden of HAE at various levels of everyday life. Diet and recreational sports in particular are triggers that should not be neglected, with one third (diet) to two thirds (recreational sports) of all patients reporting on these triggering or exacerbating factors in their everyday lives. Patients also reported severe subjective impairments caused by HAE in personal, cosmetic, or professional areas. 

As mechanical stress is known to trigger HAE attacks, increased pressure and shear forces during recreational sports can explain the induction of a local swelling. However, it has not yet been explained how consumption of food can lead to HAE attacks. Increased gas formation due to intolerances could explain increased stretching of the intestinal loops and a possibly mechanically induced triggering of an abdominal attack, e.g., through the consumption of dairy products in a lactose-intolerant patient. This mechanism can also be a possible explanation for bloating or highly water-absorbent foods such as legumes, fermented products or seeds (chia, flaxseed, psyllium). This is nowadays particularly interesting from an allergological point of view, as a wide range of vegan products with plant-based proteins (often rich in lentils, peas, beans, chickpeas) is available, that could be possible triggers in HAE, but are also representing new sources of allergens [25]. However, the extent to which IgE-associated food allergies can trigger HAE attacks can only be speculated. In the case of an IgE-mediated type 1 reaction, allergen exposure and subsequent activation of mast cells or basophils can lead to heparin release, bradykinin formation, and increased vascular permeability, while immediate-type reactions can also lead to complement activation [18, 26, 27, 28]. However, whether this is quantitatively sufficient to induce HAE attacks or pathophysiologically relevant has not been investigated. No relevant influence of atopy or allergies on HAE has been shown in previous studies [8, 18, 19]. 

National guidelines in Germany and Austria do not explicitly mention food, intolerances, or recreational sports as trigger factors, while the international guideline mentions strenuous physical activity as a possible attack trigger [1, 3, 29]. 

Nevertheless, avoidance or management of trigger factors is a crucial part of HAE counseling so that dietary habits, food allergies/intolerances, and physical activity should be taken into account when treating and advising patients with HAE. 

The limitation of this study is the small patient cohort, the possible bias due to the lack of objectivity of self-reported symptoms or suspected triggers. In addition, no additional allergological tests or exposure tests for suspected foods were performed. In order to verify the relevance and mechanisms of diet-related factors on HAE, investigations should be carried out in larger study populations and include a thorough allergological workup. 

## Ethics statement 

The study was approved by the ethical board of the Medical Faculty of the University of Leipzig (Vote Nr.: 115/19-ek). Oral and written informed consent was obtained from all participants. 

## Acknowledgment 

Clinician Scientist Program, Medical Faculty, University Hospital Leipzig. 

## Authors’ contributions 

RT and JZ were equally involved in the concept of the study; acquisition, analysis and interpretation of data and to paper writing. All authors gave their final, pre-published approval of the version and agree to be accountable for all aspects of the work. 

## Funding 

This work received no special financial support. 

## Conflict of interest 

JZ and RT were involved in clinical studies on HAE, funded by Takeda, CSL Behring, and Centogene. RT received lecture fees and JZ received travel grants from Takeda and CSL Behring.
